# Three new species of the genus *Czernyola* Bezzi, 1907 (Diptera, Clusiidae) from China

**DOI:** 10.3897/zookeys.1029.63696

**Published:** 2021-04-08

**Authors:** Shuai-Lai Yang, Xin-Ming Yin, Yu-Qiang Xi

**Affiliations:** 1 Department of Entomology, Henan Agricultural University, No. 95 Wenhua Road, Jinshui District, Zhengzhou 450003, Henan Province, China

**Keywords:** Acalyptrates, Clusiodinae, druid flies, identification key, taxonomy

## Abstract

The following three species assigned to the *Czernyola biseta* group of the genus *Czernyola* Bezzi, 1907, from China are described as new to science: *C. luteigenis***sp. nov.**, *C. planipalpis***sp. nov.**, and *C. shanxiensis***sp. nov.** A key to the known species of *Czernyola* from China is provided.

## Introduction

*Czernyola* Bezzi, 1907, belongs to the subfamily Clusiodinae, which is assigned to the family Clusiidae. The genus is characterized by the following characteristics: four or five fronto-orbitals; the third fronto-orbital seta from back inclinate and the remainder reclinate, most posterior fronto-orbital seta sometimes small or minute; mid tibia with 2 dorsal preapical setae and hind tibia with 1 dorsal preapical seta; surstylus smooth with sparse setae or setulose on the outer surface anteriorly; ejaculatory apodeme wide and flared at the end, appearing mushroom-shaped; distiphallus large, membranous and sac-like, sometimes strongly reduced or absent; hypandrial arm with a weak membranous attachment to the remainder of the hypandrium (Lonsdale and Marshall 2006). There are 59 described species distributed worldwide, of which one species is distributed in the Palaearctic Region and 20 species are distributed in the Oriental Region (Lonsdale 2017). Only one species, *Czernyola biseta* (Hendel, 1913) has been recognized from China (Hendel 1913; Hennig 1941; Frey 1960).

McAlpine (1960) divided the genus into three species groups: *Czernyola transversa*, *C. concinna*, and *C. biseta*. The *C. transversa* group is best identified on the four reclinate fronto-orbitals, united cerci, and elongate basiphallus with atrophy of the distiphallus and the ejaculatory apodeme. The *C. concinna* group is considered a weakly supported clade, based on four fronto-orbitals of approximately equal lengths and often the presence of the prescutellar acrostichal seta (Lonsdale and Marshall 2006). Sasakawa (2010) established the *C. hyalina* group, based on the length of the anterior dorsocentral seta and the shape of the surstylus.

In this paper three new species assigned to the *C. biseta* group are described based on the small hind fronto-orbitals and anterior dorsocentral seta, as well as the characteristics of the cerci and surstylus.

## Materials and methods

Genitalia preparations were made by removing and macerating the apical portion of the abdomen in glacial acetic acid, then rinsing in distilled water before storage in glycerin-filled microvials. After examination, genitalia were transferred to fresh glycerin and stored in a microvial on the pin below the specimen or moved to an ethanol tube together with the wet specimens. Specimens examined were deposited in the Entomological Museum of Henan Agricultural University (**HAU**), Zhengzhou. The general terminology follows Lonsdale and Marshall (2006). The M_1_ ratio is defined as the length of the ultimate section of wing vein M divided by the length of the penultimate section (Lonsdale and Marshall 2006).

## Taxonomy

### Key to Chinese species (males) of *Czernyola*

**Table:** 

1	Gena and palpus mostly yellow; surstylus with several apical tubercules; cerci partly unified	**2**
–	Gena and palpus at least partly brown; surstylus without apical tubercules; cerci entirely separated	**3**
2	Mesonotum entirely black; abdomen black without any yellow parts; cerci united with each other on basal half	***Czernyola biseta* (Hendel)**
–	Mesonotum dark yellow with dark brown subtriangular marking and extending to pronotum; abdomen dark brown with anterior half of tergite I yellow; cerci united with each other 1/4 to 1/2 from distal end (Fig. 3)	***Czernyola luteigenis* sp. nov.**
3	Face and gena brown or dark brown; mesonotum yellow, with brown markings at middle; pregonite with 3 strong setae of equal length (Figs 11, 12)	***Czernyola planipalpis* sp. nov.**
–	New species of Clusiidae from China Face and most of gena white; mesonotum dark brown or black; pregonite with 2 strong setae and 1 small seta (Figs 17, 18)	***Czernyola shanxiensis* sp. nov.**

### Species accounts

#### Czernyola luteigenis sp. nov.

Taxon classificationAnimaliaDipteraClusiidae

F286F217-C907-5C2B-8D2B-35441191B782

http://zoobank.org/30106DDA-5DF6-4AB2-8406-6F9393FE8CCD

Figures 1–6

##### Type material.

***Holotype***: ♂, China, Tibet, Motuo, Mt. Nanzelama, 1200 m, 23.VI.2018, leg. Qi-Cheng Yang. ***Paratypes***: 2 ♂♂, same date as holotype.

##### Diagnosis.

Head mostly yellowish; postgena with a single well-developed seta. Scutum dark yellow, mesonotum with dark brown subtriangular marking. Abdomen dark brown, anterior half of tergite I yellow. Surstylus with four distal tubercules. Pregonite with large distal lobe, distal 3/5 setulose, 1 medial seta.

##### Description.

**Male. *Body*** length 3.5 mm, wing length 2.9 mm.

**Figures 1–6.**
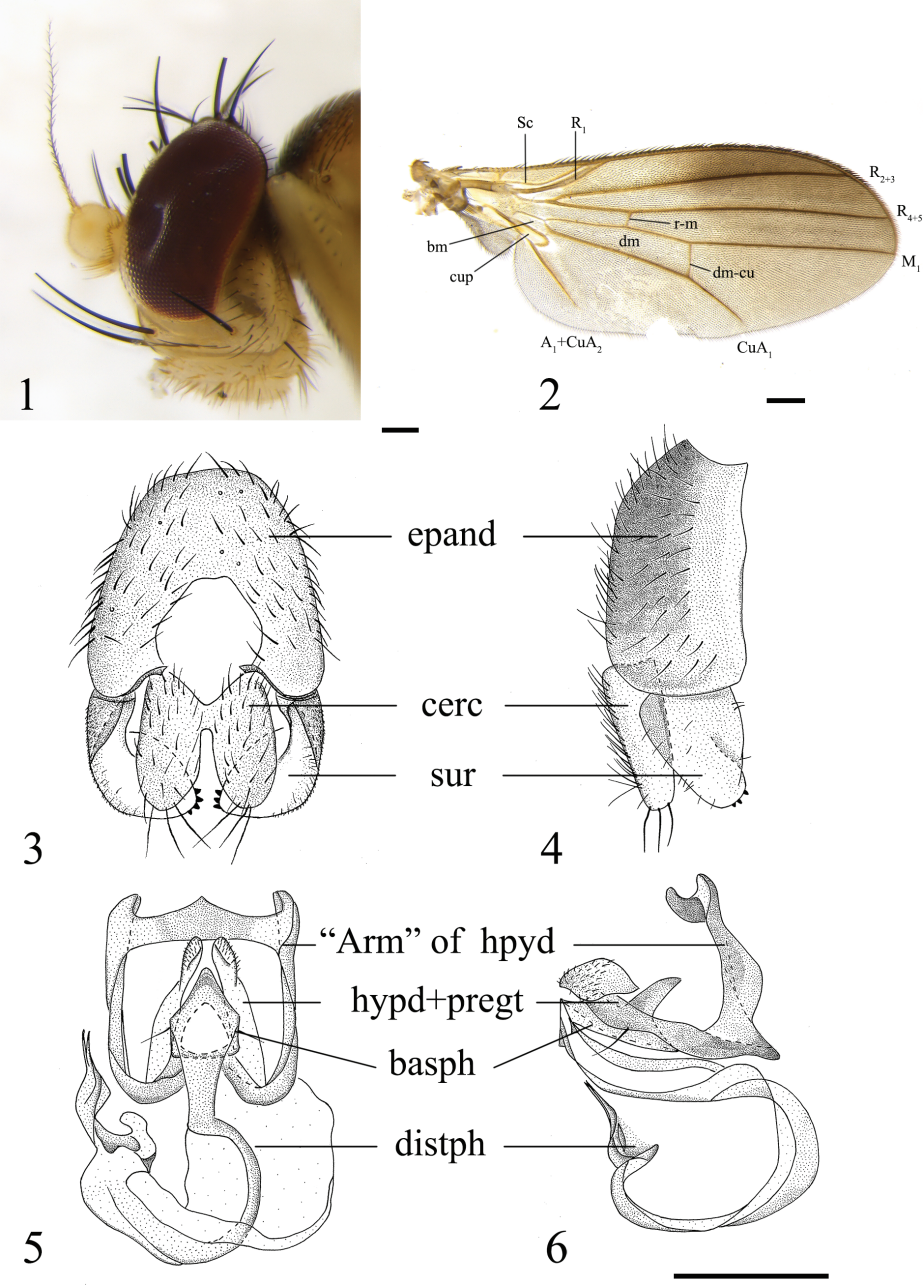
*Czernyola luteigenis* sp. nov. (male) **1** head, lateral view **2** wing **3** epandrium, cerci, and surstyli, posterior view **4** epandrium, cerci, and surstyli, lateral view **5** hypandrial complex, posterior view **6** hypandrial complex, lateral view. Scale bars: 0.1 mm. **Abbreviations**: epand – epandrium; cerc – cerci; sur – surstylus; hypd – hypandrium; pregt – pregonite; basph – basiphallus; distph – distiphallus.

***Head*** (Fig. 1) yellowish, postgena slightly brownish; frons dark yellow; occiput dark yellow in the middle and dark brown on both sides; gena ~ 1/5 as high as eye. Setae and setulae black, ocellar tubercle and anterolateral margins of the frons dark brown. Four fronto-orbitals, hind seta weak. Three genal setae and 1 well-developed seta on postgena. Antenna yellowish, first flagellomere white without infuscation. Arista sparsely short plumose. Palpus light yellow.

***Thorax*** dark yellow, pronotum dark brown anteriorly, mesonotum with dark brown subtriangular marking and anterior extend to prontum, scutellum brown and lateral margins slightly yellow. Prescutellar acrostichal seta present. One postpronotal seta, 2 notopleural setae, 1 presutural supra-alar seta, 3 dorsocentral setae, anterior seta about 1/3 length of posterior seta, 1 postsutural intra-alar seta, 2 postalar setae, 2 later scutellar setae. Prescutellar about 1/3 length of posterior dorsocentral seta. Thoracic pleura light yellow, anepisternum and posterior half of katepisternum dark brown, mediotergite brown dorsally, meron with brown tinge. One anepisternal seta, 1 katepisternal seta. Legs light yellow, coxa white. Wing (Fig. 2) dusky, more heavily infuscated on distal 1/3, along r_2+3_, the front half of all veins of the whole wing brownish and poster half of veins dark brown, M_1_ ratio: distal portion of M_1_ (the part beyond Dm-Cu) 5 × as long as Dm-Cu. Halter white.

***Abdomen*** dark brown, anterior half of the tergite I yellow; setulae and setae on abdomen black. Male genitalia (Figs 3–6): epandrium as wide as high; cerci large, oval-shaped, 2 × as high as wide, 1/4–1/2 unified at the base and the rest separated; surstylus 0.5 × length of epandrium, distal 2/3 expanded, 2 × as wide as anterior 1/3; inner and outer faces with sparse setulae, four apical tubercules. Pregonite with large distal lobe, setulose on distal 3/5 , 1 medial seta. Distiphallus long, membranous, with sclerotized bands along length, distally pointed and slightly forked.

**Female.** Unknown.

##### Distribution.

China (Tibet).

##### Etymology.

The specific name refers to the yellow gena.

##### Remarks.

This new species is somewhat similar to *Czernyola varicolor* (Sueyoshi), but differs in the surstylus bearing apical tubercules and the pregonite having obvious setae. In *C. varicolor* the surstylus has no apical tubercules, and the pregonite is without obvious medial setae (Sueyoshi 2006).

#### Czernyola planipalpis sp. nov.

Taxon classificationAnimaliaDipteraClusiidae

B74BCFFA-C738-5408-97D9-E6538D4D01FD

http://zoobank.org/3E920AC8-A909-4809-BD5D-B5E4EECC49A1

Figures 7–12

##### Type material.

***Holotype***: ♂, China, Yunnan, Xishuangbanna, Mengla County, Menglun, 556 m, 08.VII.2016, leg. Xiao-Li Li . ***Paratypes***: 2 ♂♂, China, Tibet, Motuo, Beibeng, 886 m, 10.VII.2017, leg. Qi-Cheng Yang.

##### Diagnosis.

Head brown to darkish brown; first flagellomere entirely white or dark brown. Scutum yellow with brown shield-like marking at middle. Surstylus without tubercles. Pregonite thin, with 3 strong setae nearly equal in length and many microsetulae.

##### Description.

**Male. *Body*** length 2.9 mm, wing length 2.4 mm.

**Figures 7–12.**
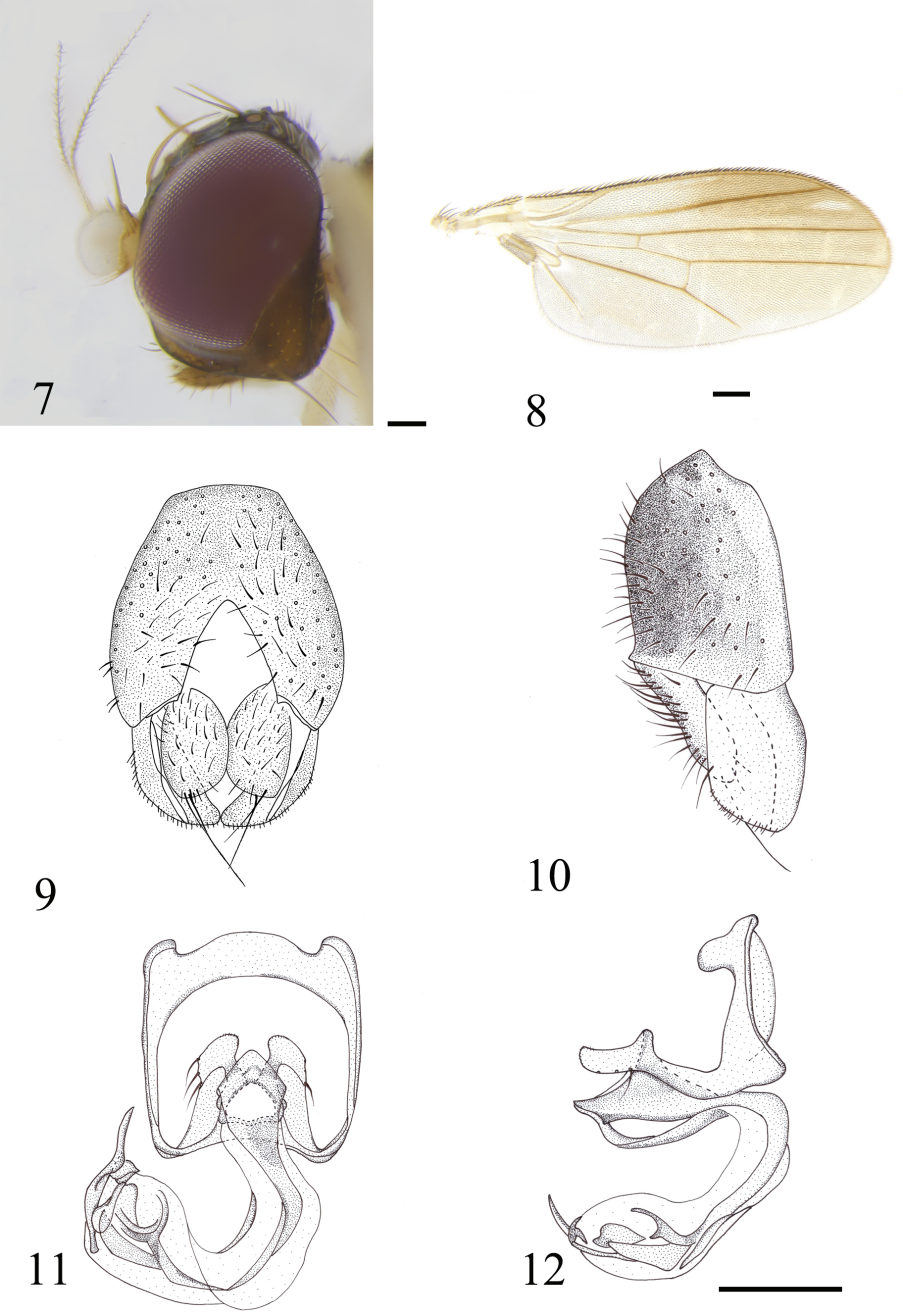
*Czernyola planipalpis* sp. nov. (male) **7** head, lateral view **8** wing **9** epandrium, cerci, and surstyli, posterior view **10** epandrium, cerci, and surstyli, lateral view **11** hypandrial complex, posterior view **12** hypandrial complex, lateral view. Scale bars: 0.1 mm.

***Head*** (Fig. 7) brown, postgena slightly lighter; frons brown; occiput darkish brown; gena ~ 1/6 as high as eye. Setae yellowish and setulae black, four fronto-orbital setae, hind seta short; genal setae and vibrissa thin. Antenna yellowish, first flagellomere yellowish white, pedicel setose with distinctly long setae dorsally. Arista sparsely short plumose. Palpus brown.

***Thorax*** yellow, pronotum with brown spot, mesonotum with large brown medial shield. Prescutellar acrostichal seta absent. One postpronotal seta, 2 notopleural setae, 2 dorsocentral setae, 1 long postsutural intra-alar seta, 1 postalar seta, 2 lateral scutellar setae, 1 apical scutellar seta, 1 long anepisternal seta, 1 katepisternal seta. Scutellum brown, microsetulae on the margin. Mediotergite brownish. Thoracic pleura whitish. Anepisternum and katepisternum brown, with several setulae. Legs yellowish white. Wing (Fig. 8) hyaline, with anterodistal infuscation, 1 hyaline small spot on r_4+5_, veins brown, M_1_ ratio: distal portion of M_1_ (the part beyond Dm-Cu) 3.5 × as long as Dm-Cu. Halter white.

***Abdomen*** dark brown, anterior 1/2 of tergite 1 yellow; setulae and setae on abdomen black. Male genitalia (Figs 9–12): epandrium brown, yellowish distally. Surstylus and cerci yellow. Surstylus with sparsely setose outer anterior face, inner face visible posteriorly, without tubercles. Cerci large, setulose, 2 long distal setae of different length. Pregonite thin, with many microchaetae, 2 strong setae nearly equal in length. Distiphallus bending and sac-like distally, consists of many sclerotized parts.

##### Dark phase

(China: Tibet): externally as described for pale phase except as follows: head much darker black; antennae dark yellow, first flagellomere dark brown. Scutum dark yellow, with brown shield shape slightly bigger and scutellum dark brown. Mediotergite much darker brown. Thoracic pleura yellowish. Anepisternum and katepisternum brown much darker. Mid and hind leg with femora and tibiae yellower.

**Female.** Unknown.

##### Distribution.

China (Yunnan, Tibet).

##### Etymology.

The specific name refers to the palpus wide, knife-shaped.

##### Remarks.

This new species is somewhat similar to *Czernyola atrifrons* (Malloch), but differs in having a yellow scutum with brown markings; in *C. atrifrons* the head is brown to dark brown, the vibrissa is small and the anterior half of tergite I is yellow (Steyskal and Sasakawa 1966).

#### Czernyola shanxiensis sp. nov.

Taxon classificationAnimaliaDipteraClusiidae

5B227F37-E500-5BE6-8B8B-A70B6D1204EE

http://zoobank.org/7BCA6566-ECD1-4E92-8D25-B51B3E54E29C

Figures 13–18

##### Type material.

***Holotype***, ♂, China, Shanxi, Fuxian, Ziwuling, 1271 m, 04.VIII.2019, leg. Qi-Cheng Yang. ***Paratypes***, 1 ♂, China, Shanxi, Huangliang, Liuyalinchang, 1120 m, 04.VIII.2019, leg. Qi-Cheng Yang; 1 ♂. China, Shanxi, Fuxian, Ziwuling, 1995 m, 07.VIII.2019, leg. Qi-Cheng Yang.

##### Diagnosis.

Head brownish or black, face and most of gena white; antenna white; palpus white. Mesonotum dark brown, with brownish lateral side, yellow to dark brown near both lateral margins. Surstylus without tubercles. Pregonite thin, with 2 strong and 1 small setae and many microchaetae.

##### Description.

**Male. *Body*** length 2.8 mm, wing length 2.5 mm.

**Figures 13–18.**
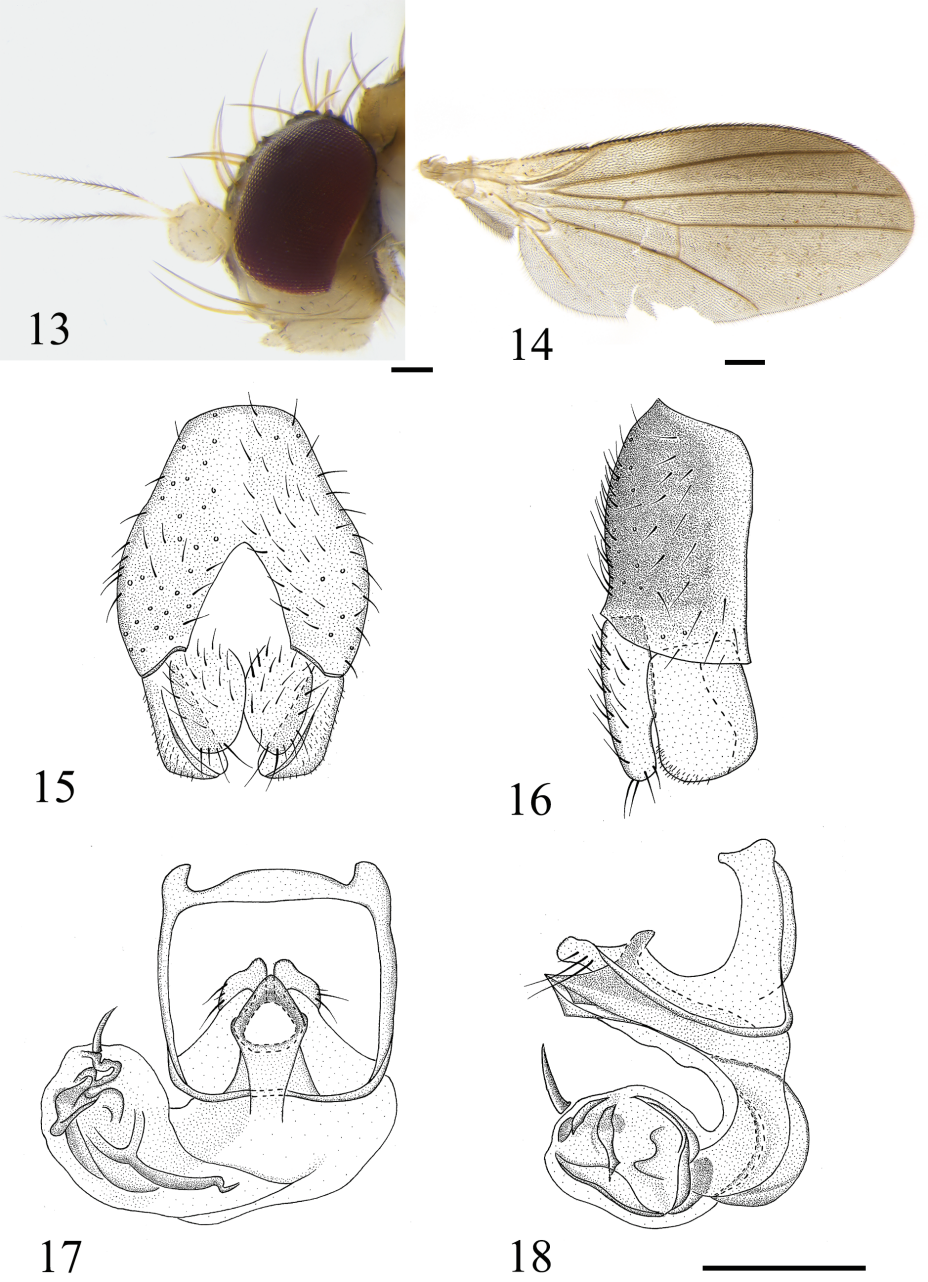
*Czernyola shanxiensis* sp. nov. (male) **13** head, lateral view **14** wing **15** epandrium, cerci, and surstyli, posterior view **16** epandrium, cerci, and surstyli, lateral view **17** hypandrial complex, posterior view **18** hypandrial complex, lateral view. Scale bars: 0.1 mm.

***Head*** (Fig. 13) brownish or black; face and gena white; gena ~ 1/8 as high as eye, with 4 setae. Setae and setulae yellowish brown, ocellar tubercle slightly bulge. Four fronto-orbitals, hind seta ½ as long as anterior seta. Ocellar seta and postvertical seta nearly equal in length, inner vertical seta as long as outer vertical seta. Vibrissa well-developed. Antenna white, arista sparsely short plumose. Palpus white.

***Thorax*** dark brown or black. Pronotum blackish dark, mesonotum dark brown with both sides brownish, margin yellow to brown, scutum much darker. Setae and setulae on thorax yellowish brown. Prescutellar acrostichal seta present. One postpronotal seta, 2 notopleural setae, 3 dorsocentral setae, anterior seta short; 1 postsutural intra-alar seta; 2 postalar setae, scutellum dark brown, 2 lateral scutellar setae, apical scutellar seta long. Thoracic pleura yellowish. Anepisternum dark yellow, with brownish spot, with 3 to 4 rows of setulae, 2 anepisternal setae, 1 katepisternal seta. Legs white yellowish. Wing (Fig. 14) dusky, more heavily infuscated on distal part along r_2+3_. Veins brown. M_1_ ratio: distal portion of M_1_ (beyond Dm-Cu) 4.3 × as long as Dm-Cu. Halter white.

***Abdomen*** dark brown, anterior half of tergite I yellow. Setulae and setae on abdomen black. Male genitalia (Figs 15–18): externally as described for *C. planipalpis* except as follows: cerci divided, large, setose and with 2 apical setae. Pregonite thin, 2 strong setae of equal lengths, 1 small seta with many microchaetae. Distiphallus sac-like, consists of many sclerotized parts.

**Female.** Unknown.

##### Distribution.

China (Shanxi).

##### Etymology.

The specific epithet refers to the type locality.

##### Remarks.

*Czernyola shanxiensis* can be separated from other species of this genus by the genitalia, as there are entirely divided cerci, the surstylus is without tubercules, and pregonite is thin, and it has 2 strong setae of equal length,1 smaller seta, and many microchaetae.

## Supplementary Material

XML Treatment for Czernyola luteigenis

XML Treatment for Czernyola planipalpis

XML Treatment for Czernyola shanxiensis
